# Prevalence and methodological quality of systematic reviews in Korean medical journals

**DOI:** 10.4178/epih.e2023017

**Published:** 2023-02-06

**Authors:** Seong Jung Kim, Mi Ah Han, Jae Hung Jung, Eu Chang Hwang, Hae Ran Kim, Sang Eun Yoon, Seo-Hee Kim, Pius Kim, So-Yeong Kim

**Affiliations:** 1 Department of Internal Medicine, Chosun University College of Medicine, Gwangju, Korea; 2 Department of Preventive Medicine, Chosun University College of Medicine, Gwangju, Korea; 3 Department of Urology, Yonsei University Wonju College of Medicine, Wonju, Korea; 4 Center of Evidence Based Medicine, Institute of Convergence Science, Yonsei University, Seoul, Korea; 5 Department of Urology, Chonnam National University Medical School, Chonnam National University Hwasun Hospital, Hwasun, Korea; 6 Department of Nursing, Chosun University College of Medicine, Gwangju, Korea; 7 Department of Public Health, Graduate School, Chosun University, Gwangju, Korea; 8 Department of Neurosurgery, Chosun University College of Medicine, Gwangju, Korea

**Keywords:** Systematic reviews, Meta-analysis, Quality assessment, Journal articles

## Abstract

This study aimed to assess and evaluate the prevalence and methodological quality of systematic reviews (SRs) published in major Korean medical journals (KMJs). The top 15 journals with the highest Korean Medical Citation Index, published between 2018 to 2021, were selected. We assessed the methodological quality of SRs using A Measurement Tool to Assess Systematic Reviews 2 (AMSTAR 2). In total, 126 SRs were included, with an average of 32 SRs being reported annually. The overall prevalence of SRs in KMJs was 2.8%, with an increase from 2.6% in 2018 to 3.4% in 2021. Overall, the methodological quality of SRs was low (9.5% low, 90.5% critically low). More than 80% of the studies adhered to critical domain items such as a comprehensive literature search and risk of bias assessment, but for items such as protocol registration and listing excluded studies and the justification for exclusion, the adherence rate was less than 15%. While the number of SRs in KMJs steadily increased, the overall confidence in the methodological quality was low to critically low. Therefore, in order to provide the best evidence for decision-making in clinical and public health areas, editors, reviewers, and authors need to pay more attention to improving the quality of SRs.

## GRAPHICAL ABSTRACT

[Fig f1-epih-45-e2023017]

## INTRODUCTION

Systematic reviews (SRs) are one of the most valuable study designs, providing the highest level of evidence for various research questions, including those for public health, and clinical research and practice. A previous study conducted in 2014 reported that more than 8,000 SRs were indexed in MEDLINE annually, with a three-fold increase over the last decade [1].

The number of SRs published worldwide has continued to rise, with Korea being one of the top countries publishing core clinical journals [2]. In addition, many Korean medical journals (KMJs) have gone global in recent years [3]. However, the prevalence and trends of SRs published in major KMJs have not been thoroughly reported.

The validity and reliability of the evidence from SRs depend on the methodology used, which is also essential for a valid interpretation and application of the findings [4]. Even though previous studies have assessed the methodological quality of SRs in specific medical fields [4-6] or countries [7,8], limited information is available on SRs in KMJs. This study assessed the prevalence of SRs in major KMJs published in recent years and evaluated the methodological quality of those SRs.

## MATERIALS AND METHODS

### Literature search strategy

We selected major KMJs from KoreaMed (https://koreamed.org), which is a service of the Korean Association of Medical Journal Editors (KAMJE). The Korean Medical Citation Index (KoMCI) is a citation database maintained by the Korean Academy of Medical Science. It indexes KoreaMed journals and publishes annual citation reports, which analyze citations of articles published in Korean journals. The KoMCI 2018 list contained 254 journals. The top 15 journals published from 2018 to 2021 with the highest KoMCIs were selected. We searched the literature in KoreaMed on October 18, 2021. Supplementary Material 1 presents a list of the selected journals and the search strategy.

### Inclusion criteria

We included SRs regardless of whether they included a metaanalysis. A study was considered to be an SR if the authors explicitly reported that they conducted it using terms such as “systematic” or “systematically.” We also considered a study to be an SR if the authors described some of the standard steps of SRs, such as the study search and study selection process, which tend to be reproducible.

We classified the remaining studies into other types of reviews (narrative and non-systematic), primary studies (randomized controlled trials, case-control studies, cross-sectional studies, etc.), and others (correspondence, editorials, commentaries, etc.). We regarded a review as a non-systematic if it did not report the method used to perform the review, such as the study search and selection.

### Study selection

Teams of two reviewers screened information from the title and abstract from each reference independently to create duplicate records, which were then calibrated, and disagreements, if any, were resolved by discussion or consultation with a third reviewer whenever necessary. For references identified as potentially eligible, we obtained the full text. Pairs of reviewers independently conducted full-text screening and resolved any discrepancies by discussion. At each stage, we conducted calibration exercises to increase reviewers’ understanding and improve consistency among them.

### Data extraction

We extracted data using a pre-piloted data extraction form after the calibration exercises. Teams of two reviewers extracted the following data independently and in duplicate.

(1) Study characteristics: first author, number of authors, number of affiliations, international collaborative authorship, publication year, language of publication, type of question, study design of primary studies, number of primary studies, number of participants, type of intervention/exposure, type of outcome, and meta-analysis.

(2) Methodological quality assessment: We used A Measurement Tool to Assess Systematic Reviews 2 (AMSTAR 2) [9] to evaluate the methodological quality of the included SRs. The AMSTAR 2 checklist consists of a total of 16 items, including 7 critical and 9 non-critical items. SRs can be categorized based on critical weakness as high (none or 1 non-critical weakness), moderate (more than 1 non-critical weakness), low (1 critical flaw with or without non-critical weaknesses), and critically low (more than 1 critical flaw with or without non-critical weaknesses) depending on the rating.

### Statistical analysis

The SRs selected from among the studies published were categorized by the type of journal articles and the year of publication. The study characteristics were presented in terms of frequencies and percentages. The proportion of reviews was measured against each item of the AMSTAR-2, classifying the methodological quality into 4 categories. All statistical analyses were performed using SAS version 9.4 (SAS Institute Inc., Cary, NC, USA).

### Ethics statement

This study was based on published studies and did not require ethical approval.

## RESULTS

### Characteristics of the included systematic reviews

In total, 4,526 articles were identified from the top 15 KMJs published between 2018 to 2021. Among them, 3,288 (72.6%) were primary studies, and 494 (10.9%) were other types of reviews. The remaining 126 (2.8%) articles were SRs and were included in this study (Table 1, Supplementary Material 2). Supplementary Material 3 presents the detailed characteristics of the SRs included.

An average of 32 (29-37) SRs were reported annually, with the prevalence of SRs increasing from 2.6% in 2018 to 3.4% in 2021 (Table 1). International collaborative studies were rare, with only 7 (5.6%) articles published collaboratively between Korean researchers and those from other countries. We identified 76 (60.3%) SRs focusing on intervention-related questions, 16 (12.7%) investigating questions related to prognosis, and 15 (11.9%) that investigated questions that pertained to diagnosis. Furthermore, 96 (76.2%) of the SRs involved a meta-analysis (Table 2).

### Methodological quality of systematic reviews

There were 9 items to which more than 70% of SRs complied (1, 4, 5, 6, 8, 9, 11, 15, 16), but 5 items with an adherence rate lower than 50% (2, 3, 7, 10, 13). Furthermore, fewer than 15% of articles adhered to items 2 and 7, which fall under critical domains (Table 3). Therefore, the overall confidence in the methodological quality of the SRs was low (9.5%) or critically low (90.5%) (Table 4).

## DISCUSSION

This study investigated the prevalence and methodological quality of SRs recently published in major KMJs. A total of 126 SRs were investigated, and the prevalence of SRs increased from 2.6% in 2018 to 3.4% in 2021. However, unlike international trends [2], only 7 SRs were conducted through international collaboration. A well-conducted international collaboration tends to improve the completeness and quality of studies [10-12]. However, studies conducted through international collaboration might be submitted to international journals, instead of journals based in Korea.

The methodological quality of SRs, as determined using AMSTAR 2, only showed low to critically low confidence. Significant contributors to low confidence were less than 50% adherence to domains, such as protocol registration (item 2), explanation of study designs selection (item 3), provision of a list of excluded studies (item 7), reporting of funding sources of primary studies (item 10), risk of bias interpretation (item 13), and failure to adhere to critical domains. Most previous studies using AMSTAR showed methodologically moderate quality overall [7,13-19], whereas studies using AMSTAR 2 showed confidence levels ranging from low to critically low [5,6,8,20-23]. Unlike AMSTAR, AMSTAR 2 does not classify quality based on a total score, but divides the items into 7 critical and 9 non-critical domains according to importance, and then classifies the quality according to each item rating [9]. Therefore, if the critical domains are not adhered to, the quality of SRs is classified as low or critically low confidence. In this study, the proportion of adherence to items 2 and 7, which are critical domains, did not even reach 15%; this may have resulted in the low confidence.

Similarly low adherence to items 2 and 7 was reported in previous studies [6,21-23]. Regarding item 2, protocol registration, only 14.3% of SRs in major KMJs registered or published their study protocol prior to conducting the SRs. Previous studies assessing the methodological quality of SRs in clinical areas have reported adherence to this item as 0% [6,22]. A written protocol with independent verification is recommended to reduce the risk of bias that may creep in during the process of conducting research [9].

In addition, adherence to item 7 (i.e., providing a list of excluded studies and justifying their exclusion), was rated as 13.5%. Similarly low adherence (0.0-6.3%) has been reported in previous studies [22,23]. Most SRs in this study described inclusion and exclusion criteria explicitly, but a list of excluded studies was not provided. Providing a complete list of potentially relevant studies would guarantee unbiased study selection and reproducibility of the study results [9,22,24]. However, the authors might not provide a complete list due to the relative importance of the list of included studies or words, and the space limitations of the journal. Therefore, all the authors’ reports, including appendices and supplementary materials, have been reviewed wherever necessary.

Item 10 (reporting funding sources of primary studies) was reported in fewer than 5% of SRs. This finding is similar to the proportions of 0.0-30.3% reported in previous studies [6,21-23,25]. Industry-funded studies sometimes show favor toward sponsors, due to which they are less likely to be published than independently funded studies [9,25,26]. Since this information may not be clearly visible in the design or methodology of a study, the funding source for each study should be well described, separately.

Although more than 80% of SRs included assessed the risk of bias (RoB; item 9), item 13 (i.e., review authors accounting for RoB in the individual studies when interpreting/discussing the results of the review) was reported completely, in fewer than 50% of studies. However, other studies published since 2020, assessing methodology and reporting quality of SRs or meta-analyses in various fields of medical treatment using AMSTAR 2, have reported satisfactory quality between 60% and 75% [6,20,22,23]. Accurately evaluating and interpreting the RoB of primary studies is important because combining low-quality primary studies increases bias, resulting in pooled estimates that are misleading [9]. Therefore, KMJs’ editors, reviewers, and researchers should pay more attention to these items in the future to increase the validity of SRs.

### Implications

Overall, the methodological quality of SRs published in major KMJs was low to critically low. Even if we were to expand the scope of our study to include other journals in Korea, it would not improve the methodological quality of SRs. Journal editors and reviewers need to be aware of these findings and use them to educate reviewers and guide authors to adhere to the standards of SRs. They also need to improve the methodological quality and ensure that SRs are conducted specifically by reviewers with relevant experience in the SR process.

Authors should be aware of methodological quality evaluation standards such as AMSTAR 2 when conducting or reporting SRs, as this would facilitate a transparent and systematic delivery of their study results. When using the results of published SRs, readers or institutions should keep in mind that the credibility of the results may vary, be alert to limitations when interpreting results or applying them in the field, and should review the quality of the SRs.

### Strengths and limitations

To the best of our knowledge, this study was the first to apply AMSTAR 2, the most widely used international evaluation standard, to evaluate the methodological quality of SRs in KMJs. In addition, the present study included the top 15 representative KMJs covering general and specialized medical areas. We complied with standard methods in this field, such as independent and duplicate reference screening, selection, and data extraction. We tried to ensure reliability and consistency by performing calibration exercises in all processes.

As limitations, due to the low to very low confidence of SRs in this study, we could not perform a comparative analysis to identify the study characteristics related to the methodological quality. We used AMSTAR 2 for a methodological quality assessment; however, this tool is primarily designed to evaluate SRs addressing intervention-related questions. Therefore, this study may not reflect the special requirements of reviews with other types of questions, such as prognosis or diagnostic performance.

The overall methodological quality of SRs in major KMJs had low to critically low confidence. Given the increasing prevalence and importance of SRs in providing the best evidence for decisionmaking in clinical and public health areas, stakeholders need to use our findings and pay more attention to improving the quality of SRs. In addition, future research on the current practices of the research community in Korea, including awareness of editors, reviewers, and authors, and authors’ guidance provided by journals should focus on providing additional evidence for improving the quality of SRs.

## Figures and Tables

**Figure f1-epih-45-e2023017:**
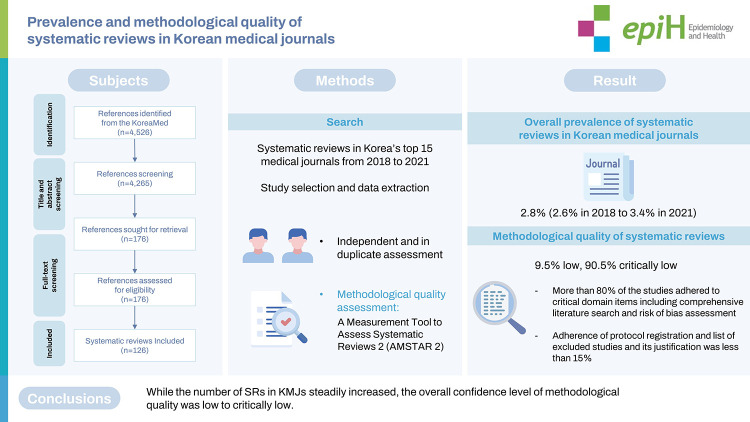


**Table 1.** Prevalence of systematic reviews in Korean medical journals

Publication year	Total	Systematic review	Other types of reviews	Primary studies	Others
2018	1,164	30 (2.6)	115 (9.9)	882 (75.8)	137 (11.8)
2019	1,211	29 (2.4)	126 (10.4)	877 (72.4)	179 (14.8)
2020	1,262	37 (2.9)	129 (10.2)	901 (71.4)	195 (15.5)
2021^1^	889	30 (3.4)	124 (13.9)	628 (70.6)	107 (12.0)
Total	4,526	126 (2.8)	494 (10.9)	3,288 (72.6)	618 (13.7)

Values are presented as number (%).

1 Including up to October 2021.

**Table 2.** Characteristics of the included studies (n=126)

Characteristics	n (%)
Publication year	
2018	30 (23.8)
2019	29 (23.0)
2020	37 (29.4)
2021	30 (23.8)
No. of authors	
2-3	29 (23.0)
4-6	61 (48.4)
≥7	36 (28.6)
No. of affiliations	
1-2	54 (42.9)
3-4	31 (24.6)
≥5	41 (32.5)
International collaborative authorship	
Korea only	69 (54.8)
Other countries only	50 (39.7)
Korea and other countries	7 (5.6)
Language of publication	
English	102 (81.0)
Korean	24 (19.1)
Type of question	
Intervention	76 (60.3)
Prognosis	16 (12.7)
Diagnosis	15 (11.9)
Prevalence	7 (5.6)
Others	12 (9.5)
Type of included studies	
Observational studies only	52 (41.3)
Randomized controlled trials only	45 (35.7)
Both	29 (23.0)
Total no. of primary studies included	
≤10	34 (27.0)
11-20	42 (33.3)
≥21	50 (39.7)
Total no. of participants included	
≤1,000	38 (30.2)
1,001-5,000	42 (33.3)
≥5,000	38 (30.2)
Not reported	8 (6.4)
Type of intervention/exposure	
Therapeutic clinical intervention	71 (56.4)
Diagnostic test	16 (12.7
Biological status	13 (10.3)
Others	26 (20.6)
Type of outcome	
Morbidity	51 (40.5)
Biophysical status	18 (14.3)
Symptoms	20 (15.9)
Mortality	10 (7.9)
Others	27 (21.4)
Meta-analysis	
No	30 (23.8)
Yes	96 (76.2)

**Table 3.** Quality assessment according to the AMSTAR 2 tool (n=126)

Items	n (%)
A1. Did the research questions and inclusion criteria for the review include the components of PICO?	125 (99.2)
A2. Did the report of the review contain an explicit statement that the review methods were established prior to the conduct of the review and did the report justify any significant deviations from the protocol?^1^	18 (14.3)
A3. Did the review authors explain their selection of the study designs for inclusion in the review?	57 (45.2)
A4. Did the review authors use a comprehensive literature search strategy?^1^	118 (93.7)
A5. Did the review authors perform study selection in duplicate?	89 (70.6)
A6. Did the review authors perform data extraction in duplicate?	92 (73.0)
A7. Did the review authors provide a list of excluded studies and justify the exclusions?^1^	17 (13.5)
A8. Did the review authors describe the included studies in adequate detail?	121 (96.0)
A9. Did the review authors use a satisfactory technique for assessing the RoB in individual studies that were included in the review?^1^	101 (80.2)
A10. Did the review authors report on the sources of funding for the studies included in the review?	5 (4.0)
A11. If meta-analysis was performed did the review authors use appropriate methods for statistical combination of results?^1,2^	81 (85.3)
A12. If meta-analysis was performed, did the review authors assess the potential impact of RoB in individual studies on the results of the meta-analysis or other evidence synthesis?^2^	41 (42.7)
A13. Did the review authors account for RoB in individual studies when interpreting/discussing the results of the review?^1^	55 (43.7)
A14. Did the review authors provide a satisfactory explanation for, and discussion of, any heterogeneity observed in the results of the review?	85 (67.5)
A15. If they performed quantitative synthesis did the review authors conduct an adequate investigation of publication bias (small study bias) and discuss its likely impact on the results of the review?^1,2^	73 (76.0)
A16. Did the review authors report any potential sources of conflict of interest, including any funding they received for conducting the review?	114 (90.5)

AMSTAR 2, A Measurement Tool to Assess Systematic Reviews 2; PICO, population, intervention, comparator, outcome; ROB, risk of bias.

1 AMSTAR 2 critical domains.

2 Did not apply to all studies, and the denominator was different (n=96).

**Table 4.** Summary quality assessment according to the AMSTAR 2 tool (n=126)

Overall confidence	n (%)
Low confidence	12 (9.5)
Critically low confidence	114 (90.5)

AMSTAR 2, A Measurement Tool to Assess Systematic Reviews 2.

## SUPPLEMENTARY MATERIALS

Supplementary Material 1. The list of selected journals and search strategy

Supplementary Material 2 Study selection process

Supplementary Material 3 General characteristics of systematic reviews included
