# Supplementary material for: Prevalence and methodological quality of systematic reviews in Korean medical journals
Source: Epidemiol Health. 2023 Feb 6;45:e2023017. doi: 10.4178/epih.e2023017 (PMC10266932; doi:10.4178/epih.e2023017)
Supplement: Supplementary Material 1. — The list of selected journals and search strategy [file epih-45-e2023017-Supplementary-1.docx]

Supplementary Appendix 1. The list of selected journals and search strategy

Supplementary Appendix 2. Study selection process

Supplementary Appendix 3. General characteristics of systematic reviews included

**Supplementary Material 1. The list of selected journals and search strategy**

The 15 journals selected based on their impact factor were *Allergy Asthma Immunol Res, Korean J Radiol, J Menopausal Med, Cancer Res Treat, J Korean Acad Nurs Adm, J Stroke, Clin Psychopharmacol Neurosci, Asian Oncol Nurs, Ultrasonography, J Bone Metab, J Korean Acad Nurs, J Korean Soc Clin Toxicol, Perspect Nurs Sci, Diabetes Metab J, and J Korean Med Sci*. We searched the literature in KoreaMed using the following key terms on October 18, 2021: (("Allergy Asthma Immunol Res"[JTI]) OR ("Korean J Radiol"[JTI]) OR ("J Menopausal Med"[JTI]) OR ("Cancer Res Treat"[JTI]) OR ("J Korean Acad Nurs Adm"[JTI]) OR ("J Stroke"[JTI]) OR ("Clin Psychopharmacol Neurosci"[JTI]) OR ("Asian Oncol Nurs"[JTI]) OR ("Ultrasonography"[JTI]) OR ("J Bone Metab"[JTI]) OR ("J Korean Acad Nurs"[JTI]) OR ("J Korean Soc Clin Toxicol"[JTI]) OR ("Perspect Nurs Sci"[JTI]) OR ("Diabetes Metab J"[JTI]) OR ("J Korean Med Sci"[JTI])) AND (2018:2021[DPY]).
