# Supplementary material for: Prevalence and methodological quality of systematic reviews in Korean medical journals
Source: Epidemiol Health. 2023 Feb 6;45:e2023017. doi: 10.4178/epih.e2023017 (PMC10266932; doi:10.4178/epih.e2023017)
Supplement: Supplementary Material 2 — Study selection process [file epih-45-e2023017-Supplementary-2.docx]

**Supplementary Material 2. Study selection process**

References identified

from the KoreaMed

(n = 4,526)

**Identification**

References screened as not a systematic review

(n =4,080)

References screened

(n =4,256)

**Title and abstract**

**screening**

References not retrieved

(n = 0)

References sought for retrieval

(n = 176)

**Full-text screening**

References assessed as not a systematic review

(n=50)

References assessed for eligibility

(n =176)

Systematic reviews included

(n = 126)

**Included**
