# Supplementary material for: Prevalence and methodological quality of systematic reviews in Korean medical journals
Source: Epidemiol Health. 2023 Feb 6;45:e2023017. doi: 10.4178/epih.e2023017 (PMC10266932; doi:10.4178/epih.e2023017)
Supplement: Supplementary Material 3 — General characteristics of systematic reviews included [file epih-45-e2023017-Supplementary-3.docx]

**Supplementary Material 3. General characteristics of systematic reviews included**

| First author, publication year | Language of publication | Type of question | Type of included studies | Total number of primary studies included | Total number of participants included | Primary intervention/exposure | Primary outcome | Meta-analysis |
| --- | --- | --- | --- | --- | --- | --- | --- | --- |
| Al-Hader 2019 | English | Prevalence | OS | 187 | 184922 | Therapeutic clinical intervention | Morbidity | conventional MA only |
| AlHajri 2021 | English | Intervention | RCT | 9 | 1285 | Therapeutic clinical intervention | Biophysical status | no MA conducted |
| Aliena-Valero 2021 | English | Diagnosis | OS | 134 | 6661 | Diagnostic test | Morbidity | no MA conducted |
| Ashe 2021 | English | Intervention | RCT | 11 | 723 | Health behavior | Morbidity | conventional MA only |
| Babakhanian 2018 | English | Intervention | RCT | 4 | 296 | Therapeutic clinical intervention | Symptoms | conventional MA only |
| Bae 2020 | Korean | Intervention | Both | 9 | 5900 | Therapeutic clinical intervention | Symptoms | conventional MA only |
| Becic 2018 | English | Intervention | RCT | 14 | 685 | Therapeutic clinical intervention | Biophysical status | conventional MA only |
| Bejleri 2021 | English | Diagnosis | OS | 22 | 4190 | Diagnostic test | Morbidity | no MA conducted |
| Bhatia 2020 | English | Prognosis | OS | 30 | 115 | Biological status | Mortality | no MA conducted |
| Cai 2021 | English | Intervention | RCT | 57 | 13240 | Therapeutic clinical intervention | Biophysical status | conventional MA only |
| Cha 2020 | English | Intervention | RCT | 8 | 822 | Therapeutic clinical intervention | Morbidity | network MA |
| Cha 2020 | English | Other | Both | 65 | 45942 | Others | Others | no MA conducted |
| Chae 2021 | Korean | Intervention | RCT | 17 | 1494 | Therapeutic clinical intervention | Others | conventional MA only |
| Chang 2018 | English | Intervention | RCT | 41 | 1972 | Therapeutic clinical intervention | Mortality | conventional MA only |
| Chang 2021 | English | Intervention | RCT | 16 | 897 | Therapeutic clinical intervention | Symptoms | conventional MA only |
| Cho 2020 | English | Intervention | Both | 17 | 999 | Therapeutic clinical intervention | Others | conventional MA only |
| Choi 2018 | English | Intervention | OS | 13 | 567 | Therapeutic clinical intervention | Mortality | conventional MA only |
| Choi 2018 | English | Intervention | OS | 60 | 9428076 | Health behavior | Morbidity | conventional MA only |
| Choi 2018 | Korean | Intervention | OS | 13 | 5125 | Therapeutic clinical intervention | Mortality | no MA conducted |
| Choi 2020 | English | Intervention | OS | 14 | 495 | Therapeutic clinical intervention | Mortality | conventional MA only |
| Choi 2020 | Korean | Intervention | RCT | 11 | 1153 | Therapeutic clinical intervention | Symptoms | conventional MA only |
| Chung 2019 | Korean | Intervention | OS | 23 | 24 | Therapeutic clinical intervention | Others | no MA conducted |
| Chung 2019 | English | Diagnosis | OS | 12 | 418 | Diagnostic test | Morbidity | no MA conducted |
| Chung 2019 | Korean | Other | OS | 36 | 12154 | Others | Others | conventional MA only |
| DeCagna 2019 | English | Intervention | RCT | 15 | 381 | Therapeutic clinical intervention | Morbidity | no MA conducted |
| Fusar-Poli 2021 | English | Prognosis | OS | 43 | 9484 | Biological status | Morbidity | conventional MA only |
| Ha 2020 | Korean | Intervention | OS | 7 | 1017 | Therapeutic clinical intervention | Morbidity | conventional MA only |
| Heidari 2019 | English | Prognosis | OS | 27 | 28088 | Biological status | Biophysical status | no MA conducted |
| Hong 2019 | English | Intervention | RCT | 6 | 249 | Therapeutic clinical intervention | Biophysical status | conventional MA only |
| Hsu 2018 | English | Intervention | RCT | 13 | 65812 | Therapeutic clinical intervention | Morbidity | conventional MA only |
| Hwang 2018 | Korean | Intervention | Both | 27 | 3110 | Health behavior | Others | no MA conducted |
| Hwang 2020 | Korean | Intervention | Both | 32 | 1663 | Others | Others | no MA conducted |
| Igwe 2018 | English | Intervention | RCT | 5 | 183 | Therapeutic clinical intervention | Morbidity | conventional MA only |
| Jang 2019 | English | Intervention | RCT | 23 | 723 | Health behavior | Biophysical status | conventional MA only |
| Jeon 2018 | English | Prognosis | OS | 8 | 21930 | Biological status | Morbidity | conventional MA only |
| Jung 2018 | Korean | Intervention | RCT | 8 | 855 | Therapeutic clinical intervention | Symptoms | conventional MA only |
| Kang 2019 | English | Intervention | Both | 17 | 17511 | Therapeutic clinical intervention | Mortality | conventional MA only |
| Kang 2020 | English | Prevalence | Both | 45 | 6127 | Others | Others | no MA conducted |
| Kerleroux 2021 | English | Intervention | RCT | 10 | 1941 | Therapeutic clinical intervention | Symptoms | conventional MA only |
| Khadivzadeh 2018 | English | Intervention | RCT | 3 | 225 | Therapeutic clinical intervention | Others | conventional MA only |
| Khadivzadeh 2018 | English | Intervention | RCT | 10 | 469 | Therapeutic clinical intervention | Symptoms | conventional MA only |
| Khadivzadeh 2018 | English | Intervention | RCT | 12 | 1257 | Therapeutic clinical intervention | Others | conventional MA only |
| Khaleghi 2020 | English | Intervention | Both | 32 | 609 | Therapeutic clinical intervention | Symptoms | no MA conducted |
| Khanra 2021 | English | Intervention | Both | 10 | 321844 | Therapeutic clinical intervention | Morbidity | network MA |
| Kim 2019 | Korean | Intervention | RCT | 23 | 1527 | Therapeutic clinical intervention | Symptoms | conventional MA only |
| Kim 2019 | English | Intervention | Both | 17 | 1321 | Therapeutic clinical intervention | Mortality | conventional MA only |
| Kim 2019 | English | Other | OS | 516 | NR | Others | Others | no MA conducted |
| Kim 2019 | English | Diagnosis | OS | 34 | 6196 | Diagnostic test | Biophysical status | conventional MA only |
| Kim 2019 | Korean | Intervention | RCT | 22 | 1461 | Therapeutic clinical intervention | Symptoms | conventional MA only |
| Kim 2019 | English | Prognosis | OS | 20 | 47930 | Biological status | Morbidity | conventional MA only |
| Kim 2020 | English | Diagnosis | OS | 20 | 766 | Diagnostic test | Others | conventional MA only |
| Kim 2020 | English | Diagnosis | OS | 13 | 2136 | Diagnostic test | Morbidity | conventional MA only |
| Kim 2021 | English | Prevalence | OS | 55 | 13527 | Others | Morbidity | conventional MA only |
| Kim 2021 | English | Intervention | OS | 4 | 653 | Therapeutic clinical intervention | Morbidity | conventional MA only |
| Kim 2021 | English | Intervention | Both | 11 | NR | Therapeutic clinical intervention | Biophysical status | conventional MA only |
| Kim 2021 | English | Diagnosis | OS | 16 | 4000 | Diagnostic test | Biophysical status | conventional MA only |
| Kim 2021 | Korean | Intervention | RCT | 37 | 3491 | Therapeutic clinical intervention | Morbidity | conventional MA only |
| Ko 2021 | English | Diagnosis | OS | 20 | 44513 | Diagnostic test | Others | conventional MA only |
| Kronenburg 2018 | English | Prevalence | OS | 17 | 434 | Others | Others | no MA conducted |
| Kulthanan 2019 | English | Prognosis | Both | 13 | 604 | Others | Others | no MA conducted |
| Lally 2019 | English | Other | OS | 15 | 1360 | Biological status | Biophysical status | no MA conducted |
| Lee 2018 | English | Intervention | RCT | 9 | 4034 | Therapeutic clinical intervention | Symptoms | network MA |
| Lee 2019 | English | Intervention | RCT | 9 | 750 | Therapeutic clinical intervention | Symptoms | conventional MA only |
| Lee 2019 | English | Intervention | OS | 8 | 10533 | Therapeutic clinical intervention | Morbidity | no MA conducted |
| Lee 2019 | English | Prognosis | OS | 7 | 3813 | Others | Morbidity | conventional MA only |
| Lee 2020 | English | Intervention | RCT | 30 | 117974 | Therapeutic clinical intervention | Morbidity | conventional MA only |
| Lee 2020 | English | Intervention | RCT | 37 | 1813 | Health behavior | Biophysical status | conventional MA only |
| Lee 2020 | English | Intervention | OS | 4 | 127722 | Therapeutic clinical intervention | Morbidity | conventional MA only |
| Lee 2020 | English | Intervention | RCT | 10 | 2126 | Therapeutic clinical intervention | Morbidity | conventional MA only |
| Lee 2020 | Korean | Other | OS | 30 | 7943 | Others | Others | conventional MA only |
| Lee 2021 | English | Intervention | RCT | 13 | 322 | Therapeutic clinical intervention | Symptoms | conventional MA only |
| Liao 2018 | English | Diagnosis | OS | 27 | NR | Diagnostic test | Morbidity | conventional MA only |
| Lim 2020 | Korean | Other | Both | 146 | NR | Others | Others | conventional MA only |
| Lim 2021 | English | Diagnosis | OS | 22 | 1732 | Diagnostic test | Morbidity | conventional MA only |
| Lin 2021 | English | Diagnosis | OS | 7 | 417 | Diagnostic test | Morbidity | conventional MA only |
| Malhotra 2019 | English | Prognosis | OS | 26 | 56513 | Biological status | Others | conventional MA only |
| Matusevicius 2021 | English | Prognosis | OS | 25 | 7543 | Biological status | Morbidity | conventional MA only |
| MohdTahir 2020 | English | Other | Both | 26 | 1234 | Others | Biophysical status | conventional MA only |
| Moriwaki 2021 | English | Intervention | Both | 15 | 2581 | Therapeutic clinical intervention | Mortality | conventional MA only |
| Nassar 2018 | English | Intervention | Both | 33 | 2714502 | Therapeutic clinical intervention | Morbidity | conventional MA only |
| Oh 2018 | Korean | Intervention | Both | 22 | 954 | Therapeutic clinical intervention | Symptoms | conventional MA only |
| Papadopoulos 2020 | English | Prognosis | OS | 31 | 25562 | Biological status | Morbidity | conventional MA only |
| Park 2018 | English | Intervention | Both | 30 | NR | Therapeutic clinical intervention | Symptoms | conventional MA only |
| Park 2018 | Korean | Other | OS | 15 | 4049 | Others | Others | conventional MA only |
| Park 2018 | Korean | Other | Both | 12 | 1226 | Others | Others | no MA conducted |
| Park 2020 | Korean | Intervention | OS | 30 | 1168 | Therapeutic clinical intervention | Morbidity | conventional MA only |
| Park 2021 | English | Intervention | OS | 19 | 2502 | Therapeutic clinical intervention | Morbidity | conventional MA only |
| Park 2021 | English | Diagnosis | OS | 25 | 4867 | Diagnostic test | Morbidity | conventional MA only |
| Rahmani 2018 | English | Intervention | RCT | 11 | 1052 | Therapeutic clinical intervention | Morbidity | conventional MA only |
| RashidiFakari 2020 | English | Intervention | RCT | 12 | 814 | Therapeutic clinical intervention | Morbidity | no MA conducted |
| Riazi 2019 | English | Intervention | RCT | 6 | 391 | Therapeutic clinical intervention | Biophysical status | no MA conducted |
| Rocha 2020 | English | Intervention | RCT | 9 | 394 | Therapeutic clinical intervention | Biophysical status | conventional MA only |
| Romoli 2020 | English | Intervention | Both | 18 | 7017 | Therapeutic clinical intervention | Mortality | conventional MA only |
| Roozbeh 2019 | English | Intervention | RCT | 4 | 310 | Therapeutic clinical intervention | Others | no MA conducted |
| Ryu 2018 | Korean | Intervention | RCT | 5 | 931 | Therapeutic clinical intervention | Others | conventional MA only |
| Seo 2019 | Korean | Intervention | RCT | 21 | NR | Therapeutic clinical intervention | Symptoms | conventional MA only |
| Seo 2021 | Korean | Intervention | RCT | 12 | 103855 | Therapeutic clinical intervention | Morbidity | conventional MA only |
| Shim 2018 | English | Intervention | RCT | 11 | 2522 | Therapeutic clinical intervention | Symptoms | no MA conducted |
| Shin 2020 | English | Intervention | Both | 10 | 3229 | Therapeutic clinical intervention | Morbidity | no MA conducted |
| Song 2019 | English | Prevalence | OS | 52 | 4931497 | Others | Others | conventional MA only |
| Song 2020 | English | Intervention | Both | 5 | 930 | Therapeutic clinical intervention | Biophysical status | conventional MA only |
| Song 2020 | English | Intervention | RCT | 3 | 203 | Therapeutic clinical intervention | Morbidity | conventional MA only |
| Suh 2020 | English | Diagnosis | RCT | 16 | 4080 | Diagnostic test | Morbidity | no MA conducted |
| Suh 2020 | English | Prognosis | RCT | 23 | 8387 | Diagnostic test | Others | conventional MA only |
| Sun 2021 | English | Intervention | OS | 5 | 597047 | Health behavior | Morbidity | conventional MA only |
| Tang 2020 | English | Prevalence | OS | 55 | 6327 | Others | Morbidity | conventional MA only |
| Torres-Aguila 2019 | English | Prognosis | OS | 61 | 88807 | Biological status | Morbidity | no MA conducted |
| Trimboli 2018 | English | Diagnosis | Both | 7 | 1187 | Diagnostic test | Morbidity | conventional MA only |
| Tsivgoulis 2019 | English | Prognosis | Both | 28 | 4531 | Therapeutic clinical intervention | Morbidity | conventional MA only |
| Vu 2021 | English | Prognosis | OS | 22 | 94575 | Biological status | Morbidity | conventional MA only |
| Wang 2018 | English | Intervention | Both | 8 | 1502 | Therapeutic clinical intervention | Mortality | conventional MA only |
| Wang 2020 | English | Intervention | RCT | 4 | 1024 | Therapeutic clinical intervention | Morbidity | conventional MA only |
| Wang 2021 | English | Intervention | RCT | 8 | 1488 | Therapeutic clinical intervention | Symptoms | conventional MA only |
| Yang 2020 | Korean | Other | OS | 24 | 26 | Health behavior | Morbidity | no MA conducted |
| Yang 2020 | English | Intervention | Both | 11 | 922 | Therapeutic clinical intervention | Others | conventional MA only |
| Yoon 2018 | English | Intervention | Both | 11 | 395 | Therapeutic clinical intervention | Biophysical status | conventional MA only |
| Yoon 2019 | English | Intervention | OS | 11 | 1407 | Therapeutic clinical intervention | Biophysical status | conventional MA only |
| Yoon 2020 | English | Other | Both | 33 | NR | Others | Others | no MA conducted |
| Yoon 2020 | English | Intervention | Both | 57 | NR | Therapeutic clinical intervention | Morbidity | no MA conducted |
| Yoon 2021 | English | Diagnosis | OS | 14 | 1233 | Diagnostic test | Biophysical status | conventional MA only |
| Yoon 2021 | English | Prognosis | OS | 7 | 241 | Biological status | Symptoms | conventional MA only |
| Yu 2018 | Korean | Other | OS | 49 | 13194 | Others | Others | conventional MA only |
| Yu 2020 | English | Prevalence | OS | 13 | 4041 | Biological status | Morbidity | conventional MA only |
| Zhang 2018 | English | Intervention | RCT | 11 | 1289 | Therapeutic clinical intervention | Symptoms | conventional MA only |
| Zhang 2019 | English | Prognosis | OS | 17 | 13468 | Others | Morbidity | conventional MA only |
| Zhong 2021 | English | Intervention | RCT | 93 | 504613 | Therapeutic clinical intervention | Morbidity | network MA |

MA, meta-analysis; OS, observational studies; RCT, randomized controlled trials

**References**

Al-Hader, R., Al-Robaidi, K., Jovin, T., Jadhav, A., Wechsler, L.R., Thirumala, P.D., 2019. The Incidence of Perioperative Stroke: Estimate Using State and National Databases and Systematic Review. J Stroke 21:290-301.

AlHajri, L., Ayoub, A., Ahmed, H., AlMulla, M., 2021. Effect of Vitamin K<sub>2</sub> Alone or in Combination on Various Bone Turnover Markers Amongst Postmenopausal Females. J Bone Metab 28:11-26.

Aliena-Valero, A., Baixauli-Martín, J., Torregrosa, G., Tembl, J.I., Salom, J.B., 2021. Clot Composition Analysis as a Diagnostic Tool to Gain Insight into Ischemic Stroke Etiology: A Systematic Review. J Stroke 23:327-42.

Ashe, M.C., Santos, I.K.d., Edward, N.Y., Burnett, L.A., Barnes, R., Fleig, L., Puyat, J.H., Sale, J.E.M., McKay, H.A., et al., 2021. Physical Activity and Bone Health in Men: A Systematic Review and Meta-Analysis. J Bone Metab 28:27-39.

Babakhanian, M., Ghazanfarpour, M., Kargarfard, L., Roozbeh, N., Darvish, L., Khadivzadeh, T., Dizavandi, F.R., 2018. Effect of Aromatherapy on the Treatment of Psychological Symptoms in Postmenopausal and Elderly Women: A Systematic Review and Meta-analysis. J Menopausal Med 24:127-32.

Bae, J., Shin, S., 2020. Factors Related to Persistent Postoperative Pain after Cardiac Surgery: A Systematic Review and Meta-Analysis. J Korean Acad Nurs 50:159-77.

Becic, T., Studenik, C., 2018. Effects of Omega-3 Supplementation on Adipocytokines in Prediabetes and Type 2 Diabetes Mellitus: Systematic Review and Meta-Analysis of Randomized Controlled Trials. Diabetes Metab J 42:101-16.

Bejleri, J., Jirström, E., Donovan, P., Williams, D.J., Pfeiffer, S., 2021. Diagnostic and Prognostic Circulating MicroRNA in Acute Stroke: A Systematic and Bioinformatic Analysis of Current Evidence. J Stroke 23:162-82.

Bhatia, R., Pedapati, R., Komakula, S., Srivastava, M.V.P., Vishnubhatla, S., Khurana, D., 2020. Stroke in Coronavirus Disease 2019: A Systematic Review. J Stroke 22:324-35.

Cai, X., Lin, C., Yang, W., Nie, L., Ji, L., 2021. Non-Insulin Antidiabetes Treatment in Type 1 Diabetes Mellitus: A Systematic Review and Meta-Analysis. Diabetes Metab J 45:312-25.

Cha, Y., Yoo, J.-I.L., Kim, J.-T., Park, C.-H., Choy, W., Ha, Y.-C., Koo, K.-H., 2020. Disadvantage during Perioperative Period of Total Hip Arthroplasty Using the Direct Anterior Approach:a Network Meta-Analysis. J Korean Med Sci 35:e111.

Cha, Y.H., Ha, Y.-C., Park, K.-S., Yoo, J.-I., 2020. What is the Role of Coordinators in the Secondary Fracture Prevention Program? J Bone Metab 27:187-99.

Chae, J.H., Kim, Y.S., Han, M.Y., 2021. Effects of Non-pharmacological Interventions on Cancer Patients with Sleep Disorder: A Meta-analysis. Asian Oncol Nurs 21:1-14.

Chang, C.-T., Hsieh, P.-J., Lee, H.-C., Lo, C.-H., Tam, K.-W., Loh, E.-W., 2021. Effectiveness of N-acetylcysteine in Treating Clinical Symptoms of Substance Abuse and Dependence: A Meta-analysis of Randomized Controlled Trials. Clin Psychopharmacol Neurosci 19:282-93.

Chang, J.S., Chiu, Y.F., Yu, J.C., Chen, L.T., Ch'ang, H.J., 2018. The Role of Consolidation Chemoradiotherapy in Locally Advanced Pancreatic Cancer Receiving Chemotherapy: An Updated Systematic Review and Meta-Analysis. Cancer Res Treat 50:562-74.

Cho, S.J., Kim, H.S., Suh, C.H., Park, J.E., 2020. Radiological Recurrence Patterns after Bevacizumab Treatment of Recurrent High-Grade Glioma: A Systematic Review and Meta-Analysis. Korean J Radiol 21:908-18.

Choi, E.H., Kim, M.J., Lee, E.N., 2020. A Meta-Analysis on the Effects of Mind-Body Therapy on Patients with Irritable Bowel Syndrome. J Korean Acad Nurs 50:385-400.

Choi, H.S., Jeong, B.K., Kang, K.M., Jeong, H., Song, J.H., Ha, I.B., Kwon, O.-Y., 2020. Tumor Control and Overall Survival after Stereotactic Body Radiotherapy for Pulmonary Oligometastases from Colorectal Cancer: A Meta-Analysis. Cancer Res Treat 52:1188-98.

Choi, M.W., Ko, D.R., Kong, T., Choa, M.H., You, J.S., Chung, S.P., 2018. Comparison of Silymarin, Penicillin, N-acetylcysteine in Patient with Amatoxin Poisoning: A Systematic Review. J Korean Soc Clin Toxicol 16:33-41.

Choi, S.H., Kim, J.W., Kim, J.H., Kim, K.W., 2018. Efficacy and Safety of Microwave Ablation for Malignant Renal Tumors: An Updated Systematic Review and Meta-Analysis of the Literature Since 2012. Korean J Radiol 19:938-49.

Choi, Y.J., Myung, S.K., Lee, J.H., 2018. Light Alcohol Drinking and Risk of Cancer: A Meta-Analysis of Cohort Studies. Cancer Res Treat 50:474-87.

Chung, H.J., Ahn, S.H., 2019. Relationship between Organizational Culture and Job Satisfaction among Korean Nurses: A Meta-Analysis. J Korean Acad Nurs Adm 25:157-66.

Chung, J.S., Choa, M.H., Chung, S.P., Park, I.C., 2019. Systematic Review of Vitamin B12 Regimen for Patient with Subacute Combined Degeneration of the Spinal Cord Following Nitrous Oxide Abuse. J Korean Soc Clin Toxicol 17:79-85.

Chung, S.R., Choi, Y.J., Suh, C.H., Lee, J.H., Baek, J.H., 2019. Diffusion-weighted Magnetic Resonance Imaging for Predicting Response to Chemoradiation Therapy for Head and Neck Squamous Cell Carcinoma: A Systematic Review. Korean J Radiol 20:649-61.

De Cagna, F., Fusar-Poli, L., Damiani, S., Rocchetti, M., Giovanna, G., Mori, A., Politi, P., Brondino, N., 2019. The Role of Intranasal Oxytocin in Anxiety and Depressive Disorders: A Systematic Review of Randomized Controlled Trials. Clin Psychopharmacol Neurosci 17:1-11.

Fusar-Poli, L., Rodolico, A., Sturiale, S., Carotenuto, B., Natale, A., Arillotta, D., Siafis, S., Signorelli, M.S., Aguglia, E., 2021. Second-to-Fourth Digit Ratio (2D:4D) in Psychiatric Disorders: A Systematic Review of Case-control Studies. Clin Psychopharmacol Neurosci 19:26-45.

Ha, J.-Y., Ban, S.-H., Lee, H.-J., Lee, M., 2020. Effects of First Assisted Reproductive Technologies on Anxiety and Depression among InfertileWomen: A Systematic Review and Meta-Analysis. J Korean Acad Nurs 50:369-84.

Heidari, M., Ghodusi, M., Rezaei, P., Kabirian Abyaneh, S., Sureshjani, E.H., Sheikhi, R.A., 2019. Sexual Function and Factors Affecting Menopause: A Systematic Review. J Menopausal Med 25:15-27.

Hong, H., Hahn, S., Choi, Y., Jang, M.J., Kim, S., Lee, J.H., Kim, H.S., 2019. Evaluation of Propofol in Comparison with Other General Anesthetics for Surgery in Children Younger than 3 Years: a Systematic Review and Meta-Analysis. J Korean Med Sci 34:e124.

Hsu, C.Y., Chiu, S.W., Hong, K.S., Saver, J.L., Wu, Y.L., Lee, J.D., Lee, M., Ovbiagele, B., 2018. Folic Acid in Stroke Prevention in Countries without Mandatory Folic Acid Food Fortification: A Meta-Analysis of Randomized Controlled Trials. J Stroke 20:99-109.

Hwang, J.-H., Seok, Y., Park, H.G., Lee, J.Y., 2020. A Systematic Review of School-bullying Interventions for Children andAdolescents in Korea. Perspect Nurs Sci 17:12-27.

Hwang, J.h., Choi, H., Jeong, H.J., Kim, C., Woo, Y., 2018. A Systematic Review of Interventions with Low-Income School-Age Children and Adolescents. Perspect Nurs Sci 15:92-106.

Igwe, S.C., Brigo, F., 2018. Does Melatonin and Melatonin Agonists Improve the Metabolic Side Effects of Atypical Antipsychotics?: A Systematic Review and Meta-analysis of Randomized Controlled Trials. Clin Psychopharmacol Neurosci 16:235-45.

Jang, J.E., Cho, Y., Lee, B.W., Shin, E.S., Lee, S.H., 2019. Effectiveness of Exercise Intervention in Reducing Body Weight and Glycosylated Hemoglobin Levels in Patients with Type 2 Diabetes Mellitus in Korea: A Systematic Review and Meta-Analysis. Diabetes Metab J 43:302-18.

Jeon, H., Min, J., Kim, D.K., Seo, H., Kim, S., Kim, Y.S., 2018. Women with Endometriosis, Especially Those Who Conceived with Assisted Reproductive Technology, Have Increased Risk of Placenta Previa: Meta-analyses. J Korean Med Sci 33:e234.

Jung, Y., Kim, J., Park, D.A., 2018. Effectiveness of Telemonitoring Intervention in Children and Adolescents with Asthma: A Systematic Review and Meta-Analysis. J Korean Acad Nurs 48:389-406.

Kang, D., Shim, S., Cho, J., Lim, H.K., 2020. Systematic Review of Studies Assessing the Health-Related Quality of Life of Hepatocellular Carcinoma Patients from 2009 to 2018. Korean J Radiol 21:633-46.

Kang, W.S., Shin, I.S., Pyo, J.S., Ahn, S., Chung, S., Ki, Y.J., Seok, J., Park, C.Y., Lee, S., 2019. Prognostic Accuracy of Massive Transfusion, Critical Administration Threshold, and Resuscitation Intensity in Assessing Mortality in Traumatic Patients with Severe Hemorrhage: a Meta-Analysis. J Korean Med Sci 34:e318.

Kerleroux, B., Janot, K., Hak, J.F., Kaesmacher, J., Hassen, W.B., Benzakoun, J., Oppenheim, C., Herbreteau, D., Ifergan, H., et al., 2021. Mechanical Thrombectomy in Patients with a Large Ischemic Volume at Presentation: Systematic Review and Meta-Analysis. J Stroke 23:358-66.

Khadivzadeh, T., Abdolahian, S., Ghazanfarpour, M., Kargarfard, L., Dizavandi, F.R., Khorsand, I., 2018a. A Systematic Review and Meta-analysis on the Effect of Herbal Medicine to Manage Sleep Dysfunction in Peri- and Postmenopause. J Menopausal Med 24:92-99.

Khadivzadeh, T., Najafi, M.N., Ghazanfarpour, M., Irani, M., Dizavandi, F.R., Shariati, K., 2018b. Aromatherapy for Sexual Problems in Menopausal Women: A Systematic Review and Meta-analysis. J Menopausal Med 24:56-61.

Khadivzadeh, T., Najafi, M.N., Kargarfard, L., Ghazanfarpour, M., Dizavandi, F.R., Khorsand, I., 2018c. Effect of Fennel on the Health Status of Menopausal Women: A Systematic and Meta-analysis. J Menopausal Med 24:67-74.

Khaleghi, A., Zarafshan, H., Vand, S.R., Mohammadi, M.R., 2020. Effects of Non-invasive Neurostimulation on Autism Spectrum Disorder: A Systematic Review. Clin Psychopharmacol Neurosci 18:527-52.

Khanra, D., Mukherjee, A., Deshpande, S., Khan, H., Kathuria, S., Kella, D., Padmanabhan, D., 2021. A Network Meta-Analysis Comparing Osteoporotic Fracture among Different Direct Oral Anticoagulants and Vitamin K Antagonists in Patients with Atrial Fibrillation. J Bone Metab 28:139-50.

Kim, D.W., Jang, H.Y., Kim, K.W., Shin, Y., Park, S.H., 2019a. Design Characteristics of Studies Reporting the Performance of Artificial Intelligence Algorithms for Diagnostic Analysis of Medical Images: Results from Recently Published Papers. Korean J Radiol 20:405-10.

Kim, D.W., Suh, C.H., Kim, K.W., Pyo, J., Park, C., Jung, S.C., 2019b. Technical Performance of Two-Dimensional Shear Wave Elastography for Measuring Liver Stiffness: A Systematic Review and Meta-Analysis. Korean J Radiol 20:880-93.

Kim, H.J., Cho, S.J., Baek, J.H., 2021. Comparison of Thermal Ablation and Surgery for Low-Risk Papillary Thyroid Microcarcinoma: A Systematic Review and Meta-Analysis. Korean J Radiol 22:1730-41.

Kim, J.-W., Han, S.C., Jo, H.D., Cho, S.-W., Kim, J.Y., 2021. Regional and Chronological Variation of Chemosensory Dysfunction in COVID-19: a Meta-Analysis. J Korean Med Sci 36:e40.

Kim, J., Chung, H.S., Choi, M.K., Roh, Y.K., Yoo, H.J., Park, J.H., Kim, D.S., Yu, J.M., Moon, S., 2019. Association between Serum Selenium Level and the Presence of Diabetes Mellitus: A Meta-Analysis of Observational Studies. Diabetes Metab J 43:447-60.

Kim, J.Y., Suh, Y.J., Han, K., Choi, B.W., 2021. Reliability of Coronary Artery Calcium Severity Assessment on Non-Electrocardiogram-Gated CT: A Meta-Analysis. Korean J Radiol 22:1034-43.

Kim, J.Y., Suh, Y.J., Han, K., Kim, Y.J., Choi, B.W., 2020. Cardiac CT for Measurement of Right Ventricular Volume and Function in Comparison with Cardiac MRI: A Meta-Analysis. Korean J Radiol 21:450-61.

Kim, K., Lee, J., 2019. Effects of Reminiscence Therapy on Depressive Symptoms in Older Adults with Dementia: A Systematic Review and Meta-Analysis. J Korean Acad Nurs 49:225-40.

Kim, M.-E., Jun, J.H., Hur, M.-H., 2019. Effects of Aromatherapy on Sleep Quality: A Systematic Review and Meta-Analysis. J Korean Acad Nurs 49:655-76.

Kim, P.H., Choi, S.H., Kim, J.H., Park, S.H., 2019. Comparison of Radioembolization and Sorafenib for the Treatment of Hepatocellular Carcinoma with Portal Vein Tumor Thrombosis: A Systematic Review and Meta-Analysis of Safety and Efficacy. Korean J Radiol 20:385-98.

Kim, P.H., Suh, C.H., Kim, H.S., Kim, K.W., Kim, D.Y., Lee, E.Q., Aizer, A.A., Guenette, J.P., Huang, R.Y., 2021. Immune Checkpoint Inhibitor with or without Radiotherapy in Melanoma Patients with Brain Metastases: A Systematic Review and Meta-Analysis. Korean J Radiol 22:584-95.

Kim, T.-H., Woo, S., Han, S., Suh, C.H., Ghafoor, S., Hricak, H., Vargas, H.A., 2020. The Diagnostic Performance of the Length of Tumor Capsular Contact on MRI for Detecting Prostate Cancer Extraprostatic Extension: A Systematic Review and Meta-Analysis. Korean J Radiol 21:684-94.

Kim, Y., Kim, B., Kim, H., 2021. Effects of Non-pharmacological Intervention on Depression among Patients with Lung Cancer: A Systematic Review and Meta-Analysis. Asian Oncol Nurs 21:140-54.

Ko, M.J., Park, D.A., Kim, S.H., Ko, E.S., Shin, K.H., Lim, W., Kwak, B.S., Chang, J.M., 2021. Accuracy of Digital Breast Tomosynthesis for Detecting Breast Cancer in the Diagnostic Setting: A Systematic Review and Meta-Analysis. Korean J Radiol 22:1240-52.

Kronenburg, A., van den Berg, E., van Schooneveld, M.M., Braun, K.P.J., Calviere, L., van der Zwan, A., Klijn, C.J.M., 2018. Cognitive Functions in Children and Adults with Moyamoya Vasculopathy: A Systematic Review and Meta-Analysis. J Stroke 20:332-41.

Kulthanan, K., Subchookul, C., Hunnangkul, S., Chularojanamontri, L., Tuchinda, P., 2019. Factors Predicting the Response to Cyclosporin Treatment in Patients With Chronic Spontaneous Urticaria: A Systematic Review. Allergy Asthma Immunol Res 11:736-55.

Lally, J., Sahl, A.B., Murphy, K.C., Gaughran, F., Stubbs, B., 2019. Serum Prolactin and Bone Mineral Density in Schizophrenia: A Systematic Review. Clin Psychopharmacol Neurosci 17:333-42.

Lee, A., Kim, T.H., Lee, H.H., Kim, Y.S., Enkhbold, T., Lee, B., Park, Y.J., Song, K., 2018. Therapeutic Approaches to Atrophic Vaginitis in Postmenopausal Women: A Systematic Review with a Network Meta-analysis of Randomized Controlled Trials. J Menopausal Med 24:1-10.

Lee, D.-s., Kim, H.-s., Choi, S.-o., Kim, E.-m., 2021. The Effects of Exercise Intervention for Post-Operative Breast Cancer Patients in Korea: A Systemic Review and Meta-Analysis of Randomized Controlled Trials. Asian Oncol Nurs 21:74-87.

Lee, E.N., Choe, S.Y., Choi, E.H., Lee, M.J., 2019. Effects of Parity and Breast Feeding Duration on the Risk of Osteoporosis in Postmenopausal Korean Women: A Systematic Review and Meta-Analysis. J Menopausal Med 25:100-07.

Lee, G., Kim, S., Yu, H., 2020. Parental Factors Associated with Smartphone Overuse in Preschoolers: A Systematic Review and Meta-Analysis. J Korean Acad Nurs 50:349-68.

Lee, J., Song, J.U., 2020. The Clinical Efficacy of Pulmonary Hypertension-Specific Agents in Idiopathic Pulmonary Fibrosis: Systematic Review and Meta-Analysis of Randomized Controlled Clinical Trials. J Korean Med Sci 35:e48.

Lee, S., Yoo, J.I., Lee, Y.K., Park, J.W., Won, S., Yeom, J., Im, J.W., Lim, S.M., Ha, Y.C., et al., 2020. Risk of Osteoporotic Fracture in Patients with Breast Cancer: Meta-Analysis. J Bone Metab 27:27-34.

Lee, S.E., Lee, J.H., Kim, H.J., Lee, B.J., Cho, S.H., Price, D., Morice, A.H., Song, W.J., 2019. Inhaled Corticosteroids and Placebo Treatment Effects in Adult Patients With Cough: A Systematic Review and Meta-analysis. Allergy Asthma Immunol Res 11:856-70.

Lee, S.H., Chae, Y.R., 2020. Characteristics of Aerobic Exercise as Determinants of Blood Pressure Control in Hypertensive Patients: A Systematic Review and Meta-Analysis. J Korean Acad Nurs 50:740-56.

Lee, Y.-K., Lee, E.-G., Kim, H.Y., Lee, Y., Lee, S.-M., Suh, D.-C., Yoo, J.-I., Lee, S., 2020. Osteoporotic Fractures of the Spine, Hip, and Other Locations after Adjuvant Endocrine Therapy with Aromatase Inhibitors in Breast Cancer Patients: a Meta-analysis. J Korean Med Sci 35:e403.

Lee, Y., Yoon, B.H., Lee, S., Chung, Y.K., Lee, Y.K., 2019. Risk of Osteoporotic Fractures after Thyroid-stimulating Hormone Suppression Therapy in Patients with Thyroid Cancer. J Bone Metab 26:45-50.

Liao, X.L., Wei, J.B., Li, Y.Q., Zhong, J.H., Liao, C.C., Wei, C.Y., 2018. Functional Magnetic Resonance Imaging in the Diagnosis of Locally Recurrent Prostate Cancer: Are All Pulse Sequences Helpful? Korean J Radiol 19:1110-18.

Lim, J.Y., Kim, E.J., Kim, S., 2020. Analysis of Variables related to Entrepreneurial Intention of University Students in Korea: Derivation of Research Variables to Promote Entrepreneurial Intention of Nursing Students. J Korean Acad Nurs Adm 26:340-53.

Lim, S.J., Kim, M., Suh, C.H., Kim, S.Y., Shim, W.H., Kim, S.J., 2021. Diagnostic Yield of Diffusion-Weighted Brain Magnetic Resonance Imaging in Patients with Transient Global Amnesia: A Systematic Review and Meta-Analysis. Korean J Radiol 22:1680-89.

Lin, Z., Lin, R., Wu, H., Wu, L., Zeng, J., Xu, J., Dong, F., 2021. Elastography for the differential diagnosis of malignant versus benign testicular lesions: a meta-analysis. Ultrasonography 40:465-73.

Malhotra, K., Ahmed, N., Filippatou, A., Katsanos, A.H., Goyal, N., Tsioufis, K., Manios, E., Pikilidou, M., Schellinger, P.D., et al., 2019. Association of Elevated Blood Pressure Levels with Outcomes in Acute Ischemic Stroke Patients Treated with Intravenous Thrombolysis: A Systematic Review and Meta-Analysis. J Stroke 21:78-90.

Matusevicius, M., Cooray, C., Rand, V.-M., Nunes, A.P., Moreira, T., Tassi, R., Egido, J.A., Ollikainen, J., Bigliardi, G., et al., 2021. Stroke Etiology and Outcomes after Endovascular Thrombectomy: Results from the SITS Registry and a Meta-Analysis. J Stroke 23:388-400.

Mohd Tahir, N.A., Saffian, S.M., Islahudin, F.H., Abdul Gafor, A.H., Makmor-Bakry, M., 2020. A Meta-Analysis on the Performance of Cystatin C- versus Creatinine-based eGFR Equations in Predicting Vancomycin Clearance. J Korean Med Sci 35:e306.

Moriwaki, T., Gosho, M., Sugaya, A., Yamada, T., Yamamoto, Y., Hyodo, I., 2021. Optimal Maintenance Strategy for First-Line Oxaliplatin-Containing Therapy with or without Bevacizumab in Patients with Metastatic Colorectal Cancer: A Meta-Analysis. Cancer Res Treat 53:703-13.

Nassar, Y., Richter, S., 2018. Proton-pump Inhibitor Use and Fracture Risk: An Updated Systematic Review and Meta-analysis. J Bone Metab 25:141-51.

Oh, P.J., Kim, Y.L., 2018. Effectiveness of Non-Pharmacologic Interventions in Chemotherapy Induced Peripheral Neuropathy: A Systematic Review and Meta-Analysis. J Korean Acad Nurs 48:123-42.

Papadopoulos, A., Palaiopanos, K., Protogerou, A.P., Paraskevas, G.P., Tsivgoulis, G., Georgakis, M.K., 2020. Left Ventricular Hypertrophy and Cerebral Small Vessel Disease: A Systematic Review and Meta-Analysis. J Stroke 22:206-24.

Park, H.-J., Son, H.G., 2020. The Effects of Depression Intervention Programs for Breast Cancer Patients in Korea : A Systematic Review and Meta-Analysis. Asian Oncol Nurs 20:150-59.

Park, H.W., Cho, M.H., Bae, S.H., Lee, R., Kim, K.S., 2021. Incidence of Postnatal CMV Infection among Breastfed Preterm Infants: a Systematic Review and Meta-analysis. J Korean Med Sci 36:e84.

Park, S.-y., Kang, B.J., 2021. Combination of shear-wave elastography with ultrasonography for detection of breast cancer and reduction of unnecessary biopsies: a systematic review and meta-analysis. Ultrasonography 40:318-32.

Park, S., Lee, T., 2018. Factors Influencing Korean Nurses' Intention to Stay: A Systematic Review and Meta-analysis. J Korean Acad Nurs Adm 24:139-48.

Park, S.C., Choi, M.Y., Choi, J., Park, E., Tchoe, H.J., Suh, J.K., Kim, Y.H., Won, S.H., Chung, Y.C., et al., 2018. Comparative Efficacy and Safety of Long-acting Injectable and Oral Second-generation Antipsychotics for the Treatment of Schizophrenia: A Systematic Review and Meta-analysis. Clin Psychopharmacol Neurosci 16:361-75.

Park, S.Y., Shin, H., Cho, Y., Kim, S., 2018. Effectiveness of Interventions for Workplace Bullying among Nurses: A Systematic Review. J Korean Acad Nurs Adm 24:339-51.

Rahmani, Y., Chaleh, K.C., Shahmohammadi, A., Safari, S., 2018. Effect of Herbal Medicine on Vaginal Epithelial Cells: A Systematic Review and Meta-analysis. J Menopausal Med 24:11-16.

Rashidi Fakari, F., Simbar, M., Beheshti Nasab, M., Ghazanfarpour, M., Rashidi Fakar, F., 2020. A Review of Pharmacological Treatments for Vaginal Atrophy in Postmenopausal Women in Iran. J Menopausal Med 26:104-11.

Riazi, H., Ghazanfarpour, M., Taebi, M., Abdolahian, S., 2019. Effect of Vitamin D on the Vaginal Health of Menopausal Women: A Systematic Review. J Menopausal Med 25:109-16.

Rocha, V.M.d., Faria, M.B.B., Júnior, F.d.A.d.R., Lima, C.O.G.X., Fiorelli, R.K.A., Cassiano, K.M., 2020. Use of Bisphosphonates, Calcium and Vitamin D for Bone Demineralization in Patients with Human Immunodeficiency Virus/Acquired Immune Deficiency Syndrome: A Systematic Review and Meta-Analysis of Clinical Trials. J Bone Metab 27:175-86.

Romoli, M., Paciaroni, M., Tsivgoulis, G., Agostoni, E.C., Vidale, S., 2020. Mothership versus Drip-and-Ship Model for Mechanical Thrombectomy in Acute Stroke: A Systematic Review and Meta-Analysis for Clinical and Radiological Outcomes. J Stroke 22:317-23.

Roozbeh, N., Ghazanfarpour, M., Khadivzadeh, T., Kargarfard, L., Dizavandi, F.R., Shariati, K., 2019. Effect of Lavender on Sleep, Sexual Desire, Vasomotor, Psychological and Physical Symptom among Menopausal and Elderly Women: A Systematic Review. J Menopausal Med 25:88-93.

Ryu, K., Lee, M., Kim, Y., Ban, S., Choi, M., 2018. How Does Advance Provision of Emergency Contraceptives Affect Contraceptive Use and Sexual Activity Among Adolescents? Systematic Review and Meta-Analysis. J Korean Acad Nurs 48:255-65.

Seo, E., Kim, S., 2019. Effect of Autogenic Training for Stress Response: A Systematic Review and Meta-Analysis. J Korean Acad Nurs 49:361-74.

Seo, J., Song, R., 2021. Effect of 2% Chlorhexidine Bathing on the Incidence of Hospital-Acquired Infection and Multidrug-Resistant Organisms in Adult Intensive Care Unit Patients: Systematic Review and Meta-Analysis. J Korean Acad Nurs 51:414-29.

Shim, I.H., Bahk, W.M., Woo, Y.S., Yoon, B.H., 2018. Pharmacological Treatment of Major Depressive Episodes with Mixed Features: A Systematic Review. Clin Psychopharmacol Neurosci 16:376-82.

Shin, Y.H., Shin, W.C., Kim, J.W., 2020. Effect of Osteoporosis Medication on Fracture Healing: An Evidence Based Review. J Bone Metab 27:15-26.

Song, J.U., Lee, J., Park, H.K., Suh, G.Y., Jeon, K., 2020. Incidence of Hypotension after Discontinuation of Norepinephrine or Arginine Vasopressin in Patients with Septic Shock: a Systematic Review and Meta-Analysis. J Korean Med Sci 35:e8.

Song, L., Luo, X., Jiang, Q., Chen, Z., Zhou, L., Wang, D., Chen, A., 2020. Vitamin D Supplementation is Beneficial for Children with Autism Spectrum Disorder: A Meta-analysis. Clin Psychopharmacol Neurosci 18:203-13.

Song, M., Latorre, G., Ivanovic-Zuvic, D., Camargo, M.C., Rabkin, C.S., 2019. Autoimmune Diseases and Gastric Cancer Risk: A Systematic Review and Meta-Analysis. Cancer Res Treat 51:841-50.

Suh, C.H., Jung, S.C., Kim, B., Cho, S.J., Woo, D.C., Oh, W.Y., Lee, J.G., Kim, K.W., 2020a. Neuroimaging in Randomized, Multi-Center Clinical Trials of Endovascular Treatment for Acute Ischemic Stroke: A Systematic Review. Korean J Radiol 21:42-57.

Suh, C.H., Kim, H.S., Jung, S.C., Choi, C.G., Kim, S.J., Kim, K.W., 2020b. Optimized Image-Based Surrogate Endpoints in Targeted Therapies for Glioblastoma: A Systematic Review and Meta-Analysis of Phase III Randomized Controlled Trials. Korean J Radiol 21:471-82.

Sun, H., Chang, Q., Liu, Y.-S., Jiang, Y.-T., Gong, T.-T., Ma, X.-X., Zhao, Y.-H., Wu, Q.-J., 2021. Adherence to Cancer Prevention Guidelines and Endometrial Cancer Risk: Evidence from a Systematic Review and Dose-Response Meta-analysis of Prospective Studies. Cancer Res Treat 53:223-32.

Tang, W.K., Wang, L., Kwok Chu Wong, G., Ungvari, G.S., Yasuno, F., Tsoi, K.K.F., Kim, J.S., 2020. Depression after Subarachnoid Hemorrhage: A Systematic Review. J Stroke 22:11-28.

Torres-Aguila, N.P., Carrera, C., MuiÃ±o, E., Cullell, N., CÃ¡rcel-MÃ¡rquez, J., Gallego-Fabrega, C., GonzÃ¡lez-SÃ¡nchez, J., Bustamante, A., Delgado, P., et al., 2019. Clinical Variables and Genetic Risk Factors Associated with the Acute Outcome of Ischemic Stroke: A Systematic Review. J Stroke 21:276-89.

Trimboli, P., Giovanella, L., 2018. Reliability of core needle biopsy as a second-line procedure in thyroid nodules with an indeterminate fine-needle aspiration report: a systematic review and meta-analysis. Ultrasonography 37:121-28.

Tsivgoulis, G., Katsanos, A.H., KÃ¶hrmann, M., Caso, V., Perren, F., Palaiodimou, L., Deftereos, S., Giannopoulos, S., Ellul, J., et al., 2019. Duration of Implantable Cardiac Monitoring and Detection of Atrial Fibrillation in Ischemic Stroke Patients: A Systematic Review and Meta-Analysis. J Stroke 21:302-11.

Vu, H., Shin, Y.-J., Kong, M.-S., Kim, H.-D., 2021. Smoking and Drinking Adjusted Association between Head and Neck Cancers and Oral Health Status Related to Periodontitis: a Meta-Analysis. J Korean Med Sci 36:e98.

Wang, S.-M., Kim, N.-Y., Na, H.-R., Lim, H.K., Woo, Y.S., Pae, C.-U., Bahk, W.-M., 2021. Rapid Onset of Intranasal Esketamine in Patients with Treatment Resistant Depression and Major Depression with Suicide Ideation: A Meta-Analysis. Clin Psychopharmacol Neurosci 19:341-54.

Wang, S.-M., Woo, Y.S., Kim, N.-Y., Na, H.-R., Lim, H.K., Bahk, W.-M., 2020. Agomelatine for the Treatment of Generalized Anxiety Disorder: A Meta-Analysis. Clin Psychopharmacol Neurosci 18:423-33.

Wang, W.d., Zhang, L.h., Ni, J.Y., Jiang, X.y., Chen, D., Chen, Y.t., Sun, H.l., Luo, J.h., Xu, L.f., 2018. Radiofrequency Ablation Combined with Transcatheter Arterial Chemoembolization Therapy Versus Surgical Resection for Hepatocellular Carcinoma within the Milan Criteria: A Meta-Analysis. Korean J Radiol 19:613-22.

Yang, J., Choi, M., Choi, J., Kang, M., Jo, A., Chung, S.H., Sim, S.H., Kim, Y.J., Yang, E.J., et al., 2020. Supervised Physical Rehabilitation in the Treatment of Patients with Advanced Cancer: a Systematic Review and Meta-analysis. J Korean Med Sci 35:e242.

Yang, S.Y., Choa, M.H., You, J.S., Chung, H.S., Chung, S.P., 2020. Acute Nicotine Poisoning due to Electronic Cigarette Liquid: Systematic Review of Case Reports. J Korean Soc Clin Toxicol 18:51-56.

Yoon, B.-H., Kim, K.-C., 2020. Does Teriparatide Improve Fracture Union?: A Systematic Review. J Bone Metab 27:167-74.

Yoon, B.-H., Koh, Y.D., Yoo, J.-I., Kim, S., Lee, G.Y., Park, S.B., Ha, Y.-C., 2021. Does Bone Mineral Density Differ between Fan-Beam and Pencil-Beam?: A Meta-Analysis and Systemic Review. J Bone Metab 28:67-77.

Yoon, B.H., Lee, Y., Oh, H.J., Kim, S.H., Lee, Y.K., 2019. Influence of Thyroid-stimulating Hormone Suppression Therapy on Bone Mineral Density in Patients with Differentiated Thyroid Cancer: A Meta-analysis. J Bone Metab 26:51-60.

Yoon, C.H., Shin, I.S., Kim, M.K., 2018. Trifocal versus Bifocal Diffractive Intraocular Lens Implantation after Cataract Surgery or Refractive Lens Exchange: a Meta-analysis. J Korean Med Sci 33:e275.

Yoon, S., Kim, Y., Lee, S.-H., 2021. Does the Loudness Dependence of Auditory Evoked Potential Predict Response to Selective Serotonin Reuptake Inhibitors?: A Meta-analysis. Clin Psychopharmacol Neurosci 19:254-61.

Yoon, Y.K., Lee, J., Kim, S.I., Peck, K.R., 2020. A Systematic Narrative Review of Comprehensive Preparedness Strategies of Healthcare Resources for a Large Resurgence of COVID-19 Nationally, with Local or Regional Epidemics: Present Era and Beyond. J Korean Med Sci 35:e387.

Yu, K.H., Park, H., Jang, K.S., 2018. Factors related to Self-leadership of Korean Clinical Nurses: A Systematic Review and Meta-analysis. J Korean Acad Nurs Adm 24:410-22.

Yu, X., Xia, L., Jiang, Q., Wei, Y., Wei, X., Cao, S., 2020. Prevalence of Intracranial Aneurysm in Patients with Aortopathy: A Systematic Review with Meta-Analyses. J Stroke 22:76-86.

Zhang, X., Lan, F., Zhang, Y., Zhang, L., 2018. Chinese Herbal Medicine to Treat Allergic Rhinitis: Evidence From a Meta-Analysis. Allergy Asthma Immunol Res 10:34-42.

Zhang, Y., Ye, B., Zheng, H., Zhang, W., Han, L., Yuan, P., Zhang, C., 2019. Association Between Organic Dust Exposure and Adult-Asthma: A Systematic Review and Meta-Analysis of Case-Control Studies. Allergy Asthma Immunol Res 11:818-29.

Zhong, X.-L., Dong, Y., Xu, W., Huang, Y.-Y., Wang, H.-F., Zhang, T.-S., Sun, L., Tan, L., Dong, Q., et al., 2021. Role of Blood Pressure Management in Stroke Prevention: A Systematic Review and Network Meta-Analysis of 93 Randomized Controlled Trials. J Stroke 23:1-11.
